# Research Progress on the Correlation Between Epigenetics and Schizophrenia

**DOI:** 10.3389/fnins.2021.688727

**Published:** 2021-07-21

**Authors:** Qing Chen, Dan Li, Weifeng Jin, Yun Shi, Zhenhua Li, Peijun Ma, Jiaqi Sun, Shuzi Chen, Ping Li, Ping Lin

**Affiliations:** Clinical Laboratory, Shanghai Mental Health Center, Shanghai Jiao Tong University School of Medicine, Shanghai, China

**Keywords:** DNA methylation, non-coding RNA, schizophrenia, epigenetics, histone modification

## Abstract

**Purpose of the Review:**

Nowadays, the incidence of schizophrenia is noticeably increased. If left undiagnosed and untreated, it will lead to impaired social functions, repeated hospital admissions, decline in quality of life and life expectancy. However, the diagnosis of schizophrenia is complicated and challenging. Both genetic and environmental factors are considered as important contributors to the development and progression of this disorder. The environmental factors have been linked to changes in gene expression through epigenetic modulations, which have raised more and more research interests in recent years. This review article is to summarize the current findings and understanding of epigenetic modulation associated with pathogenesis of schizophrenia, aiming to provide useful information for further research in developing biomarkers for schizophrenia.

**Recent Findings:**

Three major types of epigenetic modulations have been described in this article. Firstly, both DNA hypermethylation and hypomethylated have been associated with schizophrenia via analyzing post-mortem brain tissues and peripheral blood of patients. Specific changes of non-coding RNAs, particularly microRNAs and long-chain non-coding RNAs, have been observed in central and peripheral samples of schizophrenia patients, indicating their significant diagnostic value for the disease, and may also potentially predict treatment response. The correlation between histone modification and schizophrenia, however, is largely unclear.

**Summary:**

Epigenetic modulations, including DNA methylation, ncRNA transcriptional regulation and histone modification, play an important role in the pathogenesis of schizophrenia. Therefore, tests of these epigenetic alterations may be utilized to assist in the diagnosis and determination of strategies of individualized treatment in clinical practice.

## Introduction

Epigenetics refers to the heritable phenotype changes that regulate gene expression through chemical modification without changing the underlying DNA sequence. These changes mainly include DNA methylation, transcription regulation of non-coding RNA (ncRNA), and histone modification. As such, epigenetics connects both environmental and genetic factors (Allis and Jenuwein, 2016). The role epigenetic factors play in schizophrenia and its potential values in assistance with disease diagnosis and monitoring has drawn increasing attentions, and the exploration of the area was further aided by increasing number of techniques available, including MassArray (Nestler et al., 2016), methylated DNA immunoprecipitation microarray (MeDIP-chip) (Tchurikov, 2005), Droplet Digital PCR (Klose and Bird, 2006), quantitative polymerase chain reaction (Bhatia et al., 2019), and next-generation sequencing approach (Ma et al., 2018).

Schizophrenia is well-known for its high heritability and complexity of disease pathophysiology. Previous research has suggested that environmental factors, which have been linked to alteration in gene expression via epigenetic modulations, have been proposed to contribute to disease mechanisms. Various loci of genome-wide significance have been associated with schizophrenia, with each individual loci shown with minor contribution to the disease mechanism, and thus, the disease is considered polygenic rather than monogenic (International Schizophrenia et al., 2009).

In this article, we review current literatures on the correlation between epigenetic modulations (including DNA methylation, ncRNA regulation, and histone modification) and schizophrenia, providing an overall picture about recent applications of molecular biological technology in the monitoring and diagnosis of schizophrenia.

## The Association Between DNA Methylation and Schizophrenia

DNA methylation is the most-studied and best-characterized epigenetic mechanism. The process mainly occurs at CpG dinucleotides. Methylation of DNA molecules constitutes that under the catalyzation of DNA methyltransferase, the methyl group from S-adenyl methionine is transferred to the fifth carbon of cytosine residue in DNA to form 5-methylcytosine (Xu, 2015). If the DNA regulatory sequences contain a large amount of 5-methylcytosine, it can block the binding of transcription factors to DNA, thus affecting gene transcription. Therefore, methylated DNA is often associated with a decrease of gene transcription activity or gene silencing (Tchurikov, 2005; Nestler et al., 2016). In addition, methyl-CpG binding proteins can also directly silence gene expression by recognizing and binding methylated DNA and recruiting transcriptional co-inhibitors (Klose and Bird, 2006).

Abnormal DNA methylation *in utero* as well as shortly before and after birth may be related to neurodevelopmental behavioral disorders such as autism and schizophrenia (Numata et al., 2012). Some schizophrenia- and autism-associated genes, such as *DLG4* (disks large homolog 4, MIM 602887), *DRD2* (dopamine receptor D2, MIM 126450), *NOS1* (nitric oxide synthase 1, MIM 163731), *NRXN1* (neurexin-1, MIM 600565), and *SOX10* (sex-determining region Y-box 10, MIM 602229), have all been shown to have age-related dynamically methylated changes throughout the entire lifetime, especially in the fetal and postnatal stages (Numata et al., 2012).

The brain-derived neurotrophic factor (BDNF) is a neurotrophic protein that can promote neuronal development and synapse formation and has the function of maintaining learning and memory. The BDNF DNA methylation in the blood can be used as a predictor of BDNF DNA methylation and gene expression in the brain, which can be used to predict behavioral susceptibility caused by early life environmental exposure. Changes in the BDNF expression and DNA methylation level are often seen in some of the early adversity related mental diseases (e.g., depression, schizophrenia, bipolar disorder, and autism), so that BDNF DNA methylation in the blood can be used as a novel biomarker for early detection of many mental diseases (Kundakovic et al., 2015).

Previous epigenome-wide association study (EWAS) has identified a variety of differentially methylated positions (DMPs) that are associated with schizophrenia, some of which were involved in immune-function-related pathways (Hannon et al., 2016). Analysis of previous EWAS has revealed 23 genes that were differentially methylated across these EWAS on schizophrenia, such as *CACNB2* and *PRKN* (Forero and Gonzalez-Giraldo, 2020). In the Chinese Han population, it has been revealed that the most significant DMPs were pinned to genes *C17orf53*, *THAP1*, and *KCNQ4* in patients with first-episode schizophrenia (Li et al., 2020). In addition, the top-ranked differentially methylated regions (DMRs) overlapped the major histocompatibility complex (MHC), which has been shown to closely associate with schizophrenia, and schizophrenia patients presented higher polygenic risk score (PRS) compared with controls (Hannon et al., 2016). Notably, both hypermethylation and hypomethylation of DNA have been detected in individuals with schizophrenia.

Specifically, DNA hypermethylation has first been detected via analysis on post-mortem brain tissue and been linked to important neurotransmission pathways. For instance, previous post-mortem studies have revealed hypermethylation of three CpG sites in serotonin receptor 2A (*HTR2A*) as well as two CpG loci in promoter of glutamic acid decarboxylase 1 (*GAD1*), which is the enzyme controling synthesis of GABA and associated with schizophrenia-risk single nucleotide polymorphism (SNP) (Tao et al., 2018). DNA methylation was not only associated with above polymorphisms, but also with mRNA expression of the 5-hydroxytryptamine receptor 2A (*HTR2A*) (Cheah et al., 2017). In addition, hypermethylation of promoter region has been reported to suppress expression of reelin (*RELN*) and glutamic acid decarboxylase (*GAD1*) in the frontal and prefrontal cortex (Akbarian et al., 1995; Abdolmaleky et al., 2005). Analysis on prefrontal cortex has demonstrated hypermethylation of schizophrenic differential methylated genes, such as *GNA13*, *CAPNS1*, and *GABPB2* (Lee and Huang, 2016). Furthermore, some of these results from post-mortem brain tissue have been confirmed in peripheral blood. For instance, one study has shown a higher DNA methylation level of *RELN* promoters in schizophrenia patients compared with controls (Nabil Fikri et al., 2017). In addition, increased methylation of promoter of serotonin receptor type-1 (*HTR1A*) has also been revealed in blood from schizophrenia patients (Carrard et al., 2011). Higher level of methylation in CpG in gene *SLC20A1* has been associated with current suicidal ideation in schizophrenia (Bani-Fatemi et al., 2020). DNA methylation of human endogenous retroviruses K (*HERV-K*) in peripheral blood derived leukocytes was elevated in first-episode schizophrenia compared with controls (Mak et al., 2019). Paranoid schizophrenia patients presented higher percentage of mCuC and lower percentage of uCmC in *LINE-1* partial methylation compared with controls (Kalayasiri et al., 2019).

In contrast, DNA hypomethylation has also been identified in brain tissue and peripheral blood of schizophrenia patients. For instance, the hypomethylation of promoter region of catechol-O-methyltransferase (*COMT*), the gene from dopaminergic pathway, has been revealed in frontal lobe in schizophrenia (Abdolmaleky et al., 2006). In another study on peripheral blood mononuclear cells (PBMC), it has been revealed that deficit schizophrenia patients presented lower DNA methylation in *MMP9* compared with non-deficit schizophrenia patients, and *MMP9* expression is positively correlated with negative symptoms in schizophrenia (Gao et al., 2018). Apart from *MMP9*, hypomethylations have also been detected in other genes, such as *AluY A3* CpG (Li et al., 2019), promoter of *GRIN2B* (Fachim et al., 2019), CpG2 and CpG3 in *TREM2* intron 1 (Yoshino et al., 2017), CpG sites in *FAM63B* and intergenic region on chromosome 16, CpG sites in *TBC1D22A* (Sugawara et al., 2018), and *COMT* (Nour El Huda et al., 2018) in schizophrenia compared with controls. Additionally, methylation of *COMT* was lower in atypical antipsychotics group compared to typical antipsychotics group (Nour El Huda et al., 2018).

It is intriguingly noticed that the epigenetic modification may be specific in different genders. In female patients with recent onset schizophrenia and those at ultrahigh risk, oxytocin receptor gene methylation was negatively correlated with anhedonia-asociality, and functional connectivity of striatal-amygdala network was negatively correlated with methylation of the oxytocin receptor gene (*OXTR*) (Bang et al., 2019), whereas in males, DNA methylation of *COMT* has been proposed to predict risk of schizophrenia (Gao et al., 2017).

The epigenetic modification may also be associated with treatments of the disease and therefore, may act as predictors for treatment response. A meta-analysis on EWAS has revealed 1048 differentially methylated positions associated with schizophrenia, some of which were exclusively present in treatment-resistant schizophrenia (Hannon et al., 2021). In a longitudinal study on leukocytes from treatment-resistant schizophrenia patients, DNA methylation at various sites has been detected following clozapine treatment (Kinoshita et al., 2017).

## The Role of ncRNAs in Schizophrenia

The non-coding RNAs (ncRNAs) are unique RNA transcripts that have been widely identified in eukaryotic genomes and play critical parts in the occurrence and development of many diseases. The ncRNAs refer to a class of RNA molecules that are not translated into proteins, including transfer RNA (tRNA), ribosomal RNA (rRNA), small interfering RNA (siRNA), small nuclear RNA (snRNA), microRNA (miRNA), and long-chain non-coding RNA (lncRNA), among which miRNAs are the most widely studied and the most closely related ncRNAs to mental disorders (Krichevsky et al., 2003). In detail, miRNAs are short sequences (18–25 nucleotides) that can bind to the 3′UTR of the target messenger RNA (mRNA), causing repression of translation or mRNA degradation, thus leading to gene silencing. Although miRNA was first discovered in Lee et al. (1993), its role in central nervous system in patients with mental disorders has not been deeply recognized until recently. In recent years, more and more evidence has shown that miRNAs are associated with the development of mental disorders (Table 1). Among them, a novel miRNA, miR-1307, was found to be dysregulated in schizophrenia via RNA-seq (Liu et al., 2018).

**TABLE 1:** Recently reported change of microRNA expression in patients with schizophrenia.

**Name**	**Expression**	**Sample type**	**Sample size**	**References**
miR-1307	**↓**	Amygdala	22 patients and 24 healthy controls	Liu et al., 2018
miR-328	↑	Post-mortem dorsolateral prefrontal cortex tissue	37 schizophrenia/schizoaffective disorder and 37 controls	Santarelli et al., 2011
miR-17-5p	↑			
miR-134	↑			
miR-652	↑			
miR-382	↑			
miR-107	↑			
miR-130b	↑	Post-mortem brain neocortex tissue	24 schizophrenia and normal controls	Burmistrova et al., 2007
miR-92a	↑	Post-mortem prefrontal dorsolateral cortex tissue	74 schizophrenia/schizoaffective disorder and 74 controls	Santarelli et al., 2019
miR-495	↑			
miR-134	↑			
miR-132	↑	Post-mortem prefrontal cortical tissue	100 control, schizophrenic, and bipolar subjects	Miller et al., 2012
miR-137	↑	Post-mortem brain tissue	510 schizophrenia and 213 controls	Lett et al., 2013
miR-223	↑	Plasma	17 schizophrenia and 17 controls	Zhao et al., 2019
miR-30e	↑	Plasma	61 schizophrenia, 62 normal controls, and 25 patients with a 6-week antipsychotic treatment course	Sun et al., 2015b
miR-181b	↑			
miR-34a	↑			
miR-346	↑			
miR-7	↑			
miR-132	**↓** (following pharmacological treatment)			
miR-181b				
miR-432				
miR-30e				
miR-132	↑	Plasma	25 schizophrenia and 13 healthy controls	Sun et al., 2015a
miR-195	↑			
miR-30e	↑			
miR-7	↑			
miR-212	↑	PBMC	25 schizophrenia and 13 healthy controls	Sun et al., 2015a
miR-34a	↑			
miR-30e	↑			
miR-122	↑	Plasma	164 schizophrenia and 187 healthy controls	Wei et al., 2015
miR-130a	↑			
miR-130b	↑			
miR-193a-3p	↑			
miR-193b	↑			
miR-502-3p	↑			
miR-652	↑			
miR-886-5p	↑			
miR-22-3p	↑	Peripheral blood	137 schizophrenia and controls	Ma et al., 2018
miR-92a-3p	↑			
miR-137	↑			
miR-1273d	↑	PBMC	82 schizophrenia and 43 healthy controls	Chen S. D. et al., 2016
miR-1303	↑			
miR-21	↑			
miR-3064-5p	↑			
miR-3131	↑			
miR-3687	↑			
miR-4428	↑			
miR-4725-3p	↑			
miR-5096	↑			
miR-21	**↓** (following antipsychotics treatment)			
miR-34a	↑	Mononuclear leukocytes	30 schizophrenia and 30 healthy controls	Lai et al., 2011
miR-449a	↑			
miR-564	↑			
miR-432	↑			
miR-548d	↑			
miR-572	↑			
miR-652	↑			
miR-195	**↓** (following olanzapine treatment)	PBMC	81 schizophrenia received 2 months olanzapine treatment and 96 healthy controls	Huang et al., 2021

Alterations of miRNA expression has been reported in studies on post-mortem brain tissue of schizophrenia patient, and upregulation of miR-328, miR-17-5p, miR-134, miR-652, miR-382, and miR-107 has been detected (Perkins et al., 2007; Santarelli et al., 2011). Analysis on brain tissue has further revealed that expression of miR-130b is located in the locus susceptible for schizophrenia (Burmistrova et al., 2007). In addition, another post-mortem study on prefrontal dorsolateral cortex has revealed the interation between miRNAs (including miR-92a, miR-495, and miR-134) and differentially expressed genes in different pathways, such as neurodevelopment pathway, in schizophrenia (Santarelli et al., 2019). Analysis on cortical tissue revealed dysregulation of miR-132 in schizophrenia, which was regulated by NMDA and could be reversed by NMDA antagonist (Miller et al., 2012). It is noteworthy that miR-137 risk genotype has been shown to strongly predict earlier onset of psychosis in schizophrenia, and is associated with reduced integrity of white matter (Lett et al., 2013). Apart from post-mortem brain tissues, both elevation and reduction has been seen in the levels of different miRNAs in peripheral blood samples of schizophrenia patients. Analysis of plasma revealed elevated expression of miR-223 in first episode schizophrenia compared with controls (Zhao et al., 2019). Additionally, elevated plasma levels of miR-30e, miR-181b, miR-34a, miR-346, miR-7, miR-132, miR-195 (Sun et al., 2015a,b), miR-122, miR-130a, miR-130b, miR-193a-3p, miR-193b, miR-502-3p, miR-652, and miR-886-5p (Wei et al., 2015) have all been detected in multiple studies, thus, a combined test of these miRNAs may be of significant diagnostic value for the disease. Analysis on peripheral blood has also revealed abnormal expression of miR-22-3p, miR-92a-3p, and miR-137 in schizophrenia (Ma et al., 2018). On the other hand, miR-132, miR-181b, miR-432, and miR-30e were decreased following pharmacological treatment (Sun et al., 2015b). In addition to plasma, peripheral blood mononuclear cells (PBMC) have also been utilized to analyze miRNAs in multiple studies. Elevated levels of miR-1273d, miR-1303, miR-21, miR-3064-5p, miR-3131, miR-3687, miR-4428, miR-4725-3p, and miR-5096 have all been demonstrated in schizophrenia compared with controls (Chen S. D. et al., 2016). Interestingly, the miRNA signature of miR-34a, miR-449a, miR-564, miR-432, miR-548d, miR-572, and miR-652 in mononuclear leukocytes has been associated with negative symptoms and cognitive performance in schizophrenia (Lai et al., 2011). Furthermore, changes of miRNA by analyzing PBMC have also been reported and summarized in meta-analysis, showing that changes of several miRNAs may be potentially valuable for diagnosis of schizophrenia (Liu et al., 2017). In the study on serum-derived exosome from first-episode, drug-naïve schizophrenia patients, a total of eleven miRNAs have been identified to be potentially capable of differentiating schizophrenia patients from controls (Du et al., 2019). Some of the miRNAs have been identified to be associated with treatment responsiveness and efficacy rather than the disease itself. For instance, a previous study has revealed a significantly decreased level of miR-195 in peripheral blood mononuclear cells of patients responding to olanzapine treatment, although its level did not vary between drug-naïve schizophrenia patients and controls (Huang et al., 2021), and serum level of miR-21 was decreased following administration of antipsychotics (Chen S. D. et al., 2016). Moreover, both baseline level and reduction rate were positively correlated with reduction rate of positive and negative syndrome scale total scores (Huang et al., 2021).

The function of lncRNAs has been appreciated in the pathogenesis of mental diseases including schizophrenia, bipolar disease, and depression, by dysregulating the expression of target genes, and some specific lncRNAs play an important role in the recovery of depressed patients with antidepressant therapy (Table 2). A screening of lncRNAs in PBMC by microarray analysis revealed up-regulation of 62 lncRNAs, and down-regulation of 63 lncRNAs in schizophrenia compared with controls (Chen S. et al., 2016). By evaluating lncRNAs in peripheral blood, it has been revealed that *FAS-AS1*, *PVT1*, *TUG1* (Safari et al., 2019), *Neat1* and *Neat2* (Li et al., 2018) were down-regulated, while *THRIL* was upregulated in schizophrenia compared with controls (Safari et al., 2019). RNA sequencing in peripheral blood from monozygotic twins discordant for schizophrenia revealed upregulation of lncRNA-AC006129.1, which is schizophrenia associated and involved in inflammatory response (Ni et al., 2020). By analyzing human amygdala samples, the level of lncRNA-AC005009.2 has been shown to increase in schizophrenia patients compared to healthy controls (Liu et al., 2018). It has also been revealed that long non-coding RNA *DGCR5* in the 22q11.2 deletion may regulate expression of genes associated with schizophrenia within the human brain (Meng et al., 2018). Importantly, machine learning on lncRNAs generated from RNA-seq data of dorsolateral prefrontal cortex showed 96% predictive accuracy for schizophrenia (Liu et al., 2021). Furthermore, the association of *FAS-AS1*, *GAS5*, *NEAT1*, and *OIP5-AS1* with schizophrenia differ between genders (Safari et al., 2019). These studies have revealed that lncRNAs may serve as useful markers for schizophrenia, and may potentially predict treatment response, although contrary result has been observed in one study in which expressions of long non-coding RNA TMEVPG1 and NRON in blood cells did not differ between schizophrenia and controls (Melbourne et al., 2018).

**TABLE 2:** Recently reported change of lncRNA expression in patients with schizophrenia.

**lncRNA**	**Expression**	**Sample type**	**Sample size**	**References**
AC005009.2	↑	Amygdala	22 patients and 24 healthy controls	Liu et al., 2018
NON-HSAT041499	↑	Peripheral blood mononuclear cells	106 patients and 48 healthy controls	Chen S. et al., 2016
NON-HSAT089447	↑			
NON-HSAT021545	↑			
*FAS-AS1*	**↓**	Peripheral blood	50 patients and 50 healthy controls	Safari et al., 2019
*PVT1*	**↓**			
*TUG1*	**↓**			
*THRIL*	↑			
*Neat1*	**↓**	Peripheral blood	Treated and untreated schizophrenia and controls	Li et al., 2018
*Neat2*	**↓**			
lncRNA-AC006129.1	↑	Peripheral blood	Monozygotic twins discordant for schizophrenia	Ni et al., 2020

## Histone Modification and Schizophrenia

Histones are highly conserved structural proteins involved in the composition of eukaryotic chromosomes, which are divided into five types: H1, H2A, H2B, H3, and H4. Histone N-terminal tails, as post-transcriptional modification sites, can undergo chemical modifications such as methylation, acetylation, phosphorylation, adenylylation, ubiquitination and adenosine diphosphate ribosylation, thus affecting gene transcription, repair, replication, and recombination. Histone methylation generally occurs on lysine and arginine residues. K4, K9, K27, K36, and K79 (referring to lysine at positions 4, 9, 27, 36, and 79, respectively) of histone H3 and K20 of H4 can all be methylated. Enhancer of zeste homolog 2 (EZH2) can induce trimethylation of histone H3 at lysine 27 (H3K27me3), and then combines with other proteins to form polycomb repressive complex 2 (PRC2), which plays a role in maintaining transcriptional program of the cells. Meanwhile, the incorporated methyl group of H3K27Me3 can also be removed by demethylase to prevent the modification from lasting (Bannister and Kouzarides, 2011).

Acetylation of histone H3 at promoters of schizophrenia-related genes *GAD78*, *TOMM70A*, *HTR2C*, and *PPM1E* has been shown to correlate with these gene expressions (Tang et al., 2011). Specifically, increase of H3-(methyl)arginine 17 has been shown to associate with decreased expression of several metabolic-associated genes in schizophrenia (Akbarian et al., 2005).

## Conclusion

Mental disorders are complex, multifactorial diseases involving chronic changes in the structure and function of neural circuits. Schizophrenia and several other mental disorders are highly hereditary (Burmeister et al., 2008), while epigenetic disorders are caused by environmental factors such as certain life experiences and stress. These environmental factors can lead to further changes in genes or genome modifications and cause long-term harm to an individual’s health, also, the epigenetic disorders can be transferred hereditarily within a family. The discussed studies have shown that persistent abnormalities in epigenetic markers have been detected in the brain tissues as well as peripheral biospecimens of schizophrenia patients, however, the results need to be further confirmed across multiple studies and the mechanisms behind need to be further investigated. A better understanding of epigenetic effects on the development and progression of schizophrenia and other mental disorders may help in the early diagnosis and differential diagnosis of these diseases, and in the development of novel therapies for schizophrenia.

## Author Contributions

QC, DL, WJ, and PLin contributed to concept of the review. QC, DL, and WJ wrote the manuscript. PLin, YS, ZL, PM, JS, SC, and PLi reviewed and edited the manuscript. PLin finalized and supervised the manuscript. All authors contributed to the article and approved the submitted version.

## Conflict of Interest

The authors declare that the research was conducted in the absence of any commercial or financial relationships that could be construed as a potential conflict of interest.
